# Health-oriented leadership, gender-differences and job satisfaction: results from a representative population-based study in Germany

**DOI:** 10.1186/s12889-023-15014-1

**Published:** 2023-01-14

**Authors:** Regina Lutz, Nicola Jungbäck, Elisabeth Wischlitzki, Hans Drexler

**Affiliations:** Institute and Outpatient Clinic of Occupational, Social and Environmental Medicine Friedrich-Alexander-Universität Erlangen-Nürnberg, Henkestraße 9-11, 91054 Erlangen, Germany

**Keywords:** Health-oriented leadership, Gender differences, Job satisfaction, Self-rated health status, Healthy leadership, Leadership style

## Abstract

**Background:**

In recent years, the topic of health-oriented leadership (HoL) has often been investigated with health-related outcomes like general health, strain, depression, and anxiety symptoms. In contrast, research which considers the gender of leaders and employees in connection to HoL as well as studies on relationships between HoL and job satisfaction, are scarce. The aim of this paper is to explore the relationships between HoL and health status assessed by employees and leaders, to analyse the relationships between HoL and job satisfaction as a non-health-related outcome for employees and leaders and to examine differences in the assessment of HoL between men and women in a representative dataset of the working population in Germany.

**Methods:**

Data were collected via an access panel as a cross-sectional survey. The quota sample included 643 German workers (managers and employees). We focused on staff-care as a core component of HoL. Statistical analyses were performed using Pearson correlations and regression analyses as well as t-tests and Mann-Whitney-U-Tests.

**Results:**

The results showed no significant differences between male and female employees or leaders in assessing HoL. Regarding HoL we found relationships between self-rated health status or job satisfaction, both for the self-rated assessment of leaders and employees.

**Conclusions:**

Our findings indicate relationships between HoL and well-being as well as job satisfaction at the workplace. For interventions of any kind, the lack of gender effects leaves a wide scope for the implementation of health-promoting measures. In particular, the findings on the relationship between HoL and job satisfaction through leaders’ self-assessment could be used for salutogenic approaches to strengthen resources in leadership trainings.

## Introduction

In order to create a health-promoting workplace and to have healthy employees, it is necessary to comply with the legal requirements. Besides, healthy employees are relevant for the success of an organization, for innovation, progress, and growth [1, 2]. Work demands, work stress and shift work are negatively correlated with the health and well-being of employees [3–5]. Consequently, absenteeism, presentism and a reduced productivity may increase [6]. Both, the direct illness costs and the indirect costs due to production losses must be taken into account [7, 8]. The maintenance of health is thus a shared responsibility of the employees and the health-oriented behaviour of the managers in the company practice [9–13]. In recent years, the study of leadership behaviour as a health-relevant influence has become increasingly important in scientific research and in company practice [9, 11, 14, 15]. Different leadership styles, such as the transformational, the authentic or the leader-member-exchange (LMX) approach, are often mentioned as the fundamental basis for keeping employees healthy [16–19]. Models of “healthy leadership” have gained importance in occupational health science [20]. Even though “healthy leadership” is discussed controversially in comparison to other leadership styles [21], health-specific or health-oriented leadership (HoL) concepts can be described as managers’ engagement for employees’ health. HoL is a systematic approach to different ways managers can influence employees to promote health. This is, for example, through the design of working conditions, communication, or the health-oriented role model of the manager [22–25]. Several studies on HoL already addressed the relationship with health-related outcomes like general health [26, 27], strain [27, 28], burnout [29], depression or anxiety [30] for employees, but rarely focused on relationships of HoL and leader health [1]. Therefore, in addition to the relationships between HoL and follower health, as a replication study, this study focuses on the question of whether there is also a relationship between HoL and leaders’ general health. Furthermore, some studies investigated HoL and non-health-related outcomes, for example employee commitment, turnover intention, or job satisfaction. So far, only a few studies focused on the relationship between HoL and job satisfaction by employee-ratings [1, 13, 31], but there is no study which examines HoL and job satisfaction from the perspective of the leaders. Therefore, it is an open question if there is a relationship between HoL as a health-specific leadership style and job satisfaction of managers.

Regarding the gender of leaders and employees, few studies focused on differences in the assessment of HoL so far [1, 27, 32–36]. Assuming that women are more likely to have a transformational or participative leadership style and men an authoritarian leadership style, it can be concluded that there might also be differences in HoL [37–39]. Previous research shows on the one hand a higher HoL in female leaders [33], other studies on the other hand did not show significant results [35, 36]. Some studies found different results in different samples [1, 27]. As the overall evidence regarding HoL and gender-differences remains contradictory and inconsistent, it is useful to get another deep insight on this topic.

The aim of this study is (1) to explore the relationships between HoL and health status rated by employees and leaders, (2) to analyze the relationships between HoL and job satisfaction as a non-health-related outcome for employees and leaders and (3) to examine differences in the assessment of HoL between men and women in a representative dataset of the working population in Germany. This work aims to shed more light to the state of research regarding the relationships between HoL and health-related as well as non-health-related outcomes and to provide a deeper insight from the leaders’ perspective. Furthermore, the aim of this work is to expand the still unclear and inconsistent state of research regarding gender effects in health-oriented leadership in terms of a replication study. From a practical perspective, we aim to present concrete suggestions based on the theoretical implications. This can be used for occupational safety and health and workplace health promotion as well as for management training. For practical implications it would be conceivable, for example, that if gender differences emerge, that these differences might be addressed in management training courses.

## Theory and hypotheses

This section contains the theoretical background of our analyses and presents the corresponding hypotheses.

### Leadership as a health factor

In recent years, leadership in connection with health factors has increasingly become the focus of scientific research. Gregersen et al. (2011) showed in a review that leadership can act as both a stressor and a resource for employees. They highlight that numerous studies have already examined the influence of leadership on the health and well-being of employees [11, 40–43]. Their findings are that transformational and employee-oriented leadership have a health-promoting effect [11]. The authors state that most studies which examined this relationship could be confirmed empirically. Moreover, a meta-analysis by Montano et al. [41] shows positive health effects, stress reduction and a lower tendency to burnout as well as an increase in well-being as a result of transformational leadership [44] or employee-oriented leadership.

Leadership can affect health in four possible ways, which are mentioned in the following: Indirect impact paths, for example (1) via working conditions or personality traits [11] or (2) directly via communication and interaction, (3) by the manager’s own experience of stress or (4) the role model effect [22–25]. Since our study focuses on health-oriented leadership, this leadership style, as well as its associations with health, job satisfaction as a non-health-related outcome, and gender differences are described below.

### Health-oriented leadership

Health-specific leadership can be described as a domain-specific leadership style that focuses on employee health. Accordingly, the leader supports employee health and shows health-supportive behaviour in order to reduce work-related demands [29, 45, 46]. By explaining additional variance in health outcomes, health-specific leadership can be differentiated from general leadership styles such as transformational, transactional leadership or LMX [26, 45]. In recent years, various approaches to capture health-specific leadership have been proposed. These are for example health- and development promoting leadership [32, 47], health-promoting leadership [12], and health-oriented leadership (HoL, [1, 14]). In our study, we relate to HoL according to Franke and Felfe (2011), Franke et al. (2014), and Pundt and Felfe (2017) [1, 14, 26]. The HoL concept by Franke et al. (2014) integrates the above-mentioned four ways of leaders’ influence on followers’ health. It encompasses both employee-directed HoL (1 and 2) and self-directed health-oriented self-leadership (4). By assuming effects of leader’s health on employees’ health the stress perspective of leaders is considered (3) [26]. An integrative approach by including the perspectives of leaders and employees (staff-care and self-care) was created. Staff-care can be understood as an external, self-care as an internal resource [26], following the COR theory [48]. A health-promoting approach to the managers’ own health (self-care) is to be seen as an important prerequisite for health-promoting leadership behaviour (staff-care) and followers’ health [49]. The Absenteeism Report 2021 [50] shows that employees are particularly dependent on staff-care by the leader in times of crisis, but it is precisely then that leaders find it difficult to lead employees in a way that promotes health due to reduced healthy self-leadership [49]. Three components in explaining the relationship between leadership and employee health are named “value”, “awareness”, and “behaviour” [1, 14, 26]. “Value” describes the importance of one’s own health and the health of employees from managers’ perspective. This also includes the design of working conditions. “Awareness” includes the perception of stressful experience and health status, as well as conditions that influence the experience. “Behaviour” implies the level of personal activity regarding health-related actions and patterns of behaviour [1]. In our study, we focus on staff-care as the external resource and core component of HoL.

### Health-oriented leadership and health

It remains evident that HoL has several health outcomes which will be described in the following (focus: staff-care). First, we report the current state of research for the employees’ samples and second, for the leaders’ samples.

**Employees.** Franke et al. (2014) reported significant relationships between staff-care (awareness, value, and behaviour) and the state of health [26]. Another study shows relationships between staff-care (awareness, promotion and risk) and self-rated health or irritation [27]. Regarding the employee surveys in geriatric care facilities, Horstmann (2018) found a significant negative relationship between HoL and burnout [29]. In the study of Santa Maria et al. (2021) HoL was also negatively related to burnout in a sample of police officers [51]. An experimental study by Klebe et al. (2021) showed a negative correlation between staff-care and follower exhaustion/strain [52]. Köppe et al. (2018) showed that the extent of leaders’ exhaustion is negatively related to staff-care behaviour assessed by their followers and that staff-care behaviour has a negative effect on employees’ somatic problems [36]. Vonderlin et al. (2020) found significant relationships between employee HoL ratings (awareness, value, behaviour) and their individual depression and anxiety symptoms [30].

HoL shows correlations with various health indicators, such as stress, anxiety, burnout risk or even depression of the employees. It can be assumed that there is also a correlation with the health status of the employees, assessed by employees. We assume that HoL influences health status. The study by Alimo-Metcalfe et al. (2008), for example, supports our assumption and shows that leadership influences followers‘ well-being [53]. However, it cannot be excluded that health status also affects HoL. Based on the fact that HoL is a domain-specific leadership style that focuses on employee health in general, we expect that leaders support employee health and show health-supportive behaviour in order to reduce work-related demands. To improve employee health, leaders pay attention to the warning signals and signs of overload of their employees, for example. By providing a positive team climate and beneficial resources, addressing health issues like presentism or reminding the employees to engage in stress prevention courses, as well as avoiding excessive overtime, leaders show health-oriented leadership. Complying to breaks and work hours potentially gives the employees more possibilities to take a rest and recover from work strain. These measures (among others) nurture on the one side employees’ awareness of stress, on the other side reduce stressful working conditions and therefore improve employee health. Thus, we hypothesize the following:

**H1a:** Health-oriented leadership (staff-care) is positively related to employees’ health status.

**Leaders.** Pundt and Felfe (2017) found no significant correlations of leaders’ psychological health risk and the components awareness and value of staff-care, but significant correlations between leaders’ psychological health risk and the behaviour component. They showed positive relationships between staff-care facet awareness and leaders’ general health and negative relationships with leaders’ strain, and work-family conflicts. In contrast, the facet behaviour showed only positive associations with leaders’ health status and negative ones with work-family conflicts [1]. Grimm et al. (2021) reported in a leader sample negative correlations between staff-care and exhaustion or job demands and positive correlations between staff-care and engagement or job resources [54]. Several studies showed negative relationships between leaders’ strain and staff care [28, 55]. To the best of our knowledge, there are no other studies reporting leaders’ general health and HoL (staff-care) assessed by leaders.

We expect, if a manager shows a high HoL towards employees, this person is already deeply concerned with health regarding work related factors. This would mean that health-oriented awareness and behavior toward employees would have to be related to the manager’s own health awareness and behavior (self-care) and subsequently also to the manager’s own state of health. Therefore, managers who show health-oriented leadership are better able to recognize warning signals of overload not solely with their employees, but first with themselves. As role models for employees, they ideally participate in stress prevention courses and make sure they do not show presentism themselves. Thus, in addition to serving as role models, they are also able to improve their own health at the same time. Based on this consideration, we expect that HoL affects leaders’ health status and hypothesize the following:

**H1b:** Health-oriented leadership (staff-care) is positively related to leaders’ health status.

### Health-oriented leadership and job satisfaction

After the previous explanations have dealt with leadership and the respective health effects, the next section is dedicated to the connection between HoL and another outcome parameter, job satisfaction.

Wofford (1971) describes job satisfaction as the sum of attitudes towards well-being at work and the corresponding framework conditions [56]. According to Locke (1976) job satisfaction is „a pleasurable or positive emotional state resulting from the appraisal of one’s job or job experiences” (p. 1304) [57]. Thus, both definitions emphasise positive attitudes towards work. Bruggemann et al. (1975) pointed out that job satisfaction can also include the negative attitudes [58]. Why leadership behaviour can support employees’ job satisfaction could be explained by the social identity theory of leadership [59]. According to this, the identification of an employee with the workgroup can be influenced by the behaviour of leaders. A stronger identification is related to a higher job satisfaction and a lower turn-over intention [60].

In terms of leadership, Walumbwa et al. (2005) showed a strong positive relationship between transformational leadership style and job satisfaction [18]. Piccolo et al. (2012) concluded that transformational leadership is one of the most important predictors of employee job satisfaction [61]. Emmerich and Rigotti (2021) also postulated positive correlations between transformational and authentic leadership on job satisfaction [17]. A recent review by Dannheim et al. (2021) primarily examined the effects of HoL interventions on the health and well-being of employees. The results also include a study that found statistically significant changes in job satisfaction after leadership interventions (short-term follow-up) [62]. Most studies in the field of HoL focus on health-related outcomes like physical or psychological health, stress or strain and not on non-health-related variables such as job satisfaction (e.g. [26, 30, 35, 36, 51, 63]). To provide a precise understanding, we divided the following sections in employees’ and leaders’ findings regarding HoL and job satisfaction, as we did in the previous chapter.

**Employees.** Pundt and Felfe (2017) reported significant correlations between job satisfaction and staff-care [1]. A study by Krick et al. (2021) found that staff-care buffered the negative effect of job demands on general health state and job satisfaction and showed a direct positive relationship of staff-care on job satisfaction [31]. Bregenzer et al. (2020) showed in their study a significant relationship from health-promoting leadership on job satisfaction in an Austrian and Slovenian sample [13]. It can be assumed that HoL is related to positive resources of employees at the workplace [54]. For example, employees with a high level of mindfulness can recognize stress signals more quickly and reflect on them or exchange about these topics with their partners, family, or colleagues. Thus, these employees will also faster identify the source of stress and can find possible solutions to reduce stress or at least communicate this to the manager. Other job resources like the opportunity to use skills, the possibility to try out new ideas or autonomy can also help employees to deal with high job demands. Through higher resources, the work environment can be evaluated more positively and there is a stronger identification with the organization. These factors are also related to job satisfaction [13]. In addition, the study of Bregenzer et al. (2020) suggests that HoL influences job satisfaction and that the reverse path seems to be unlikely [13]. Thus, we also assume that HoL affects job satisfaction. Based on the underlying mechanism that HoL is related to positive workplace resources, HoL can also support job satisfaction itself. We expect the following:

**H2a:** Health-oriented leadership (staff-care) is positively related to employees’ job satisfaction.

**Leaders.** Nothing is yet known about HoL staff-care and leaders’ job satisfaction. Previous studies only examined employee samples to explore the relationship of HoL and job satisfaction. It seems possible, that leaders who display HoL to the employees as a role-model, have therefore more resources and less stress at work. Managers who care about the health of their followers, foster resources, and offer their employees autonomy, support them in taking breaks, promote safety at work, motivate employees to attend in stress prevention courses, etc., will receive positive feedback from employees. Positive feedback and employees who show high job satisfaction can also promote job satisfaction of the manager himself. This is imaginable by a positive work climate with less complaints, a lower rate of absenteeism and in the long term in a higher loyalty and lower fluctuation. Altruistic behaviour could satisfy the giving or helping person, what might lead to a higher job satisfaction of the leaders themselves. Based on the aforementioned research gap, we postulate the following:

**H2b:** Health-oriented leadership (staff-care) is positively related to leaders’ job satisfaction.

### Gender effects in health-oriented leadership

In the last decades, several studies have been conducted addressing gender issues and leadership styles [33, 37, 38, 64–66]. It can be seen that women and men differ little in terms of the leadership skills they need, such as intelligence, but differ significantly in terms of the leadership style they use [37]. An interactive and transformational leadership style, which embodies core messages such as trust, shared vision and leading by example, is more likely to be carried out by women. The transformational attributes thus tend to correspond to feminine characteristics [39]. According to Eagly und Johnson (1990), women are inclined to have a democratic or participative leadership style [38]. Men, by contrast, are attributed a more authoritarian leadership style with instrumental, rational, and competent characteristics. They describe the male type as dominant, independent and task-oriented [37].

There are, however, only few studies on HoL that include the gender of the leader and the evidence is less clear. Vincent (2011, 2012) as well as Bader (2017) point out that gender-specific effects regarding leader as well as employees must be investigated in the future [32–34].

To provide a precise understanding, we divided the following section in leaders’ and employees’ findings regarding HoL and gender differences, as we did in the previous chapter.

**Leaders.** Vincent (2012) reported that leadership behaviour that promotes health and development is more in line with a “female” leadership style and found small significant effects [33]. Pundt and Felfe (2017) found, that female leaders report more staff-care as well as selfcare than male leaders [1]. They argue that female leaders assessing higher HoL (staff-care) reflects the women gender role [1]. According to this, female leaders are expected to demonstrate leadership behaviour that is characterized by interest in others, empathy, and caring as well as supportive behaviour [67, 68]. Köppe et al. (2018) found no gender differences regarding staff-care behaviour [36]. In a multisource study linking leader staff-care to HoL no gender effects were found either [35].

On the one hand, studies show higher staff-care in female leaders, and, on the other hand, no gender effects could have been proven by other studies. Thus, the existing evidence can be described as unclear and inconsistent.

We expect that women in a leadership position are more likely than men to exhibit health-promoting behaviors, as women are more likely to hold favorable health-related attitudes and health behaviors. Based on their gender role, women are attributed with empathy, a high level of social competence and interest in others alongside with health-oriented leadership. Being aware of signals of stress and reducing possible stressors can be seen as health-supportive behaviour. Regarding the self-reflection of female managers concerning the thematization of health-relevant aspects at the workplace (e.g., reducing stressors, supporting employees’ work-organization, reminding to take breaks, etc.), it could be assumed that women are more likely than men to consider their leadership to be health-oriented. To address this again as a replication, we postulate the following:

**H3:** Female leaders show higher health-oriented leadership (staff-care) than male leaders.

**Employees.** Examining employees’ rating of managers’ HoL (staff-care), Pundt and Felfe (2017) found no significant results related to gender [1]. The study of Klug et al. (2019) also found no correlations between gender and follower self-care, leader self-care and the dimensions awareness and promotion of staff-care (employees’ point of view) [27]. In another sample of health insurance providers differences in gender regarding staff-care dimension “risk” were found by Klug et al. (2019) [27].

Vincent (2012) reported that the leadership behaviour of female managers is assessed as more health-promoting by female employees than by male employees (medium effects) [33]. On the contrary, Pundt and Felfe (2017) found in employees’ reports that female leaders engage in more health risk behaviours than male leaders [1]. They stated that this result should be interpreted with caution. Vincent (2012) also supposed that the leadership behaviour of male managers is assessed as more health-promoting by female employees than by male employees. This research assumption could not be supported; on the one hand it shows that male managers lead male employees in a more health-promoting way than female employees. On the other hand, female managers lead female employees only slightly more health-promoting than male employees [33]. If we suppose that, in the context of a same-sex role model (for further details, see Elprana et al., 2015 [23]), a person is more positively inclined towards their own gender than towards the opposite gender, even in health-oriented leadership (e.g., supporting employees’ work-organization, engaging in stress prevention courses, reminding to take breaks; all in all: fostering a healthy work environment), female employees would have to perceive the female leader as more health-oriented than the male leader. For male employees applies the same from their perspective: they will perceive the male manager as more health-oriented than the female manager. Therefore, we expect:

**H4:** Employees (men and women) assess the health-oriented leadership (staff-care) of female managers higher than of male managers.

**H5a:** Female employees assess the health-oriented leadership (staff-care) of a female manager higher than male employees.

**H5b:** Male employees assess the health-oriented leadership (staff-care) of a male manager higher than female employees.

**Fig. 1:**
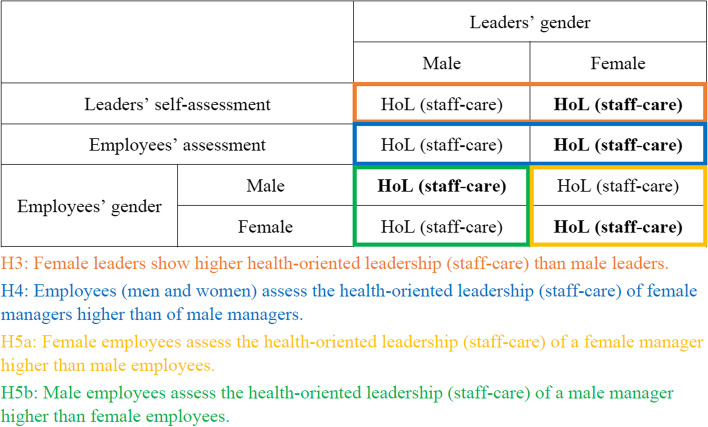
Field schema of H3, H4, H5a and H5b. *Note.* Bold-lettering for the assumed direction of hypotheses

Figure 1 displays the differences between H3, H4, H5a and H5b.

## Method

### Procedure and sample

We tested the hypotheses based on a sample that was obtained through a cross-sectional survey within the framework of the project “Healthy Working in Thuringia” (“Gesund arbeiten in Thüringen”). An online questionnaire was made available in June 2021 via an access panel. The survey was conducted as a quota sample and corresponds to the German working population in terms of gender, age, and level of education. A total of 643 German workers were available for data analysis. Since an access panel via respondi was used, no statements can be made about the response rate. The respondents were between 18 and 67 years old (*M* = 43.2; *SD* = 12.9); 49.5% were male; 0.8% had no graduation, 26.7% had a lower secondary school qualification, 34.1% had a secondary school certificate, 19.3% had a university entrance qualification and 19.1% had a university degree. The sample included both managers (*n* = 116) and employees without managerial responsibility (*n* = 523); four persons did not indicate their status and were excluded for further analyses regarding managerial responsibility. The respondents originated from a wide variety of industries. The most represented industries were medical and non-medical health, transportation and logistics, social and cultural service, and food and hospitality. 25.7% of the persons could not classify themselves in the predefined industries.

### Measures

In our survey, we used the German versions of various questionnaires and for assessing HoL, we used self-developed questions.

**Health-oriented leadership.** The draft of the questionnaire was initially developed as framework of a field-study for workplace health promotion. To enable the most economical and shortest possible option for the survey of HoL, we designed 6 items to record staff-care. The items should be suitable for the self-assessment by managers as well as for the external assessment by employees. The items for both samples were very similar, as can be seen in Table 1. As the facet awareness includes perception of stressful experience and health status, as well as conditions that influence the experience, it seemed to us - apart from economic considerations - to be the most dispensable for this setting. In the circle of the project partners, we decided to survey stress and the corresponding influencing factors via other questionnaires, which are not part of this contribution. Therefore, the facet awareness is missing in the present questionnaire.

**Table 1:** Questions for measuring health-oriented leadership

	**Employee questionnaire (English)**	**Employee questionnaire (German)**
(1)	My supervisor cares about my health.	Meine Führungskraft kümmert sich um meine Gesundheit.
(2)	My supervisor supports me in achieving the best possible work-life balance.	Meine Führungskraft unterstützt mich darin, meine Work-Life-Balance bestmöglich umzusetzen.
(3)	It is important to my supervisor to have healthy employees.	Meiner Führungskraft ist es wichtig, gesunde Beschäftigte zu haben.
(4)	My supervisor gives me the acknowledgement I deserve.	Meine Führungskraft schenkt mir die Anerkennung, die ich verdient habe.
(5)	My supervisor takes all possible measures to reduce health hazards at my workplace or to prevent them from occurring in the first place.	Meine Führungskraft trifft alle ihm/ihr möglichen Maßnahmen, um gesundheitliche Belastungen an meinem Arbeitsplatz zu reduzieren bzw. erst gar nicht entstehen zu lassen.
(6)	My supervisor cares about occupational safety in my workplace.	Meiner Führungskraft ist der Arbeitsschutz an meinem Arbeitsplatz wichtig.
	**Leader questionnaire (English)**	**Leader questionnaire (German)**
(1)	I care about the health of my employees.	Ich kümmere mich um die Gesundheit meiner Beschäftigten.
(2)	I support employees in achieving the best possible work-life balance.	Ich unterstütze die Beschäftigten darin, ihre Work-Life-Balance bestmöglich umzusetzen.
(3)	It is important to me to have healthy employees.	Mir ist es wichtig, gesunde Beschäftigte zu haben.
(4)	I give my employees the acknowledgement they deserve.	Ich schenke meinen Beschäftigten die Anerkennung, die sie verdient haben.
(5)	I take all possible measures for my employees to reduce health hazards at the workplace or to prevent them from arising in the first place.	Ich treffe für meine Beschäftigten alle möglichen Maßnahmen, um gesundheitliche Belastungen am Arbeitsplatz zu reduzieren bzw. erst gar nicht entstehen zu lassen.
(6)	Occupational safety is important to me at every workplace in the company.	Mir ist der Arbeitsschutz an jedem Arbeitsplatz im Unternehmen wichtig.

*Note.* (1), (2), (5) are derived from the behaviour-related component of HoL (Pundt and Felfe, 2017); (3), (4), (6) are derived from the value-related component of HoL

For developing adequate questions for assessing HoL in our sample we oriented ourselves in parts to the existing HoL questionnaire [1, 14]. The requirements for the questions should be as suitable as possible in the company context, e.g., for a supplementary survey in the context of a risk assessment and especially to be used in the framework of the project “Healthy working in Thuringia”. Thus, an occupational health perspective has been included. The answers can be marked on a 7-point Likert scale from *(1) completely disagree* to *(7) completely agree.*

**Job satisfaction.** To assess the participants’ job satisfaction we used the scale B.14 from the long version of the German COPSOQ Questionnaire [69, 70]. This is: “Regarding your work in general. How pleased are you with...” and consists of 7 different items such as “...your work prospects? “ or “...the way your group is run?”. On a Likert scale of 1 *(highly unsatisfied)* to 4 *(very satisfied),* a high score corresponds to a high job satisfaction. A sum value for job satisfaction was calculated from the 7 items.

**Health status.** Self-rated health status was measured with the item from the SF-36 questionnaire [71]. This is “How would you describe your state of health in general?” The answer options were slightly modified by us and were recorded on a 6-point scale from “excellent” (1) to “poor” (6), similar to the German school grading system.

### Analytic procedure

The data analysis was carried out using R Studio, based on R (version 4.1.1). First, exploratory factor analyses (EFA) were calculated for the questions on HoL (employee and leader questionnaire), as the items were self-developed based on the HoL-questionnaire and the sample was initially intended to provide an overview (hence no CFA was performed). For the items of the employee and the leader questionnaire on HoL, we assumed that they represent one factor each. The items are based on the theoretical background [14], so this assumption seemed plausible to us. We tested this assumption using the principal axis method (PAF) to account for error variance and estimated the communalities in an iterative procedure using the R package “psych” [72, 73].

The screeplot of the PAF showed the extraction of exactly one factor for HoL, both for the self-assessment of the managers and the employee questionnaire. This supports our assumption from the theoretical background. Table 2 provides information on the factor loadings according to varimax rotation and the respective corrected item-total correlations. In addition, we calculated reliability measures (Cronbach’s alpha).

**Table 2:** Factor loadings and corrected item-total correlations for employee and leader questionnaire

	**Employee questionnaire**	**Factor loadings**	**corrected item-total correlations**
(1)	My supervisor cares about my health.	.878	.851
(2)	My supervisor supports me in achieving the best possible work-life balance.	.864	.837
(3)	It is important to my supervisor to have healthy employees.	.835	.811
(4)	My supervisor gives me the acknowledgement I deserve.	.892	.864
(5)	My supervisor takes all possible measures to reduce health hazards at my workplace or to prevent them from occurring in the first place.	.932	.900
(6)	My supervisor cares about occupational safety in my workplace.	.828	.803
	**Leader questionnaire**	**Factor loadings**	**corrected item-total correlations**
(1)	I care about the health of my employees.	.698	.670
(2)	I support employees in achieving the best possible work-life balance.	.724	.695
(3)	It is important to me to have healthy employees.	.749	.699
(4)	I give my employees the acknowledgement they deserve.	.846	.777
(5)	I take all possible measures for my employees to reduce health hazards at the workplace or to prevent them from arising in the first place.	.799	.744
(6)	Occupational safety is important to me at every workplace in the company.	.783	.715

All items of the employee questionnaire had factor loadings above .80 and of the leader questionnaire above .60. The explained variance was 76% (employees) and 59% (managers), respectively. Corrected item-total correlations were above .50 for all statements and can be assumed to be a very good value [74]. For the scale job satisfaction, corrected item-total correlations were above .60 for all 7 items and can be also assumed to be a good value [74].

Subsequently, sum values were calculated for the scales. For the testing of H1 and H2, we performed Pearson correlations and multiple regression analyses. For H3, H4 and H5b we used t-tests, for H5a a Mann-Whitney-U-test. Due to multiple testing of the (sub)sample(s), we adjusted *p*-values [75]. Furthermore, we included control-variables such as age and education level. Finally, two additional, explorative moderator analyses with the “PROCESS”-function by Andrew F. Hayes were performed for the employees’ sample [76]. In this analysis, HoL was used as the predictor variable, job satisfaction or self-rated health-status used as the respective outcome variables, and leaders’ gender was used as the moderator variable.

## Results

To test H1 and H2, we first examined the respective relationships via Pearson correlations. Table 3 displays all results for the employee sample and the leader sample, including Cronbach’s α. To check whether our samples could be biased, we included age and education level as control-variables.

**Table 3:** Means, standard deviations, α, and correlations with confidence intervals for employees’ and leaders’ sample

Variable	*M*	*SD*	α	1	2	3
Employees						
1. health-oriented leadership	4.18	1.72	.94			
2. self-rated health status	3.81	1.21	–	.41**		
				[.33, .49]		
3. job satisfaction	2.89	0.61	.89	.70**	.42**	
				[.64, .74]	[.34, .50]	
4. age	43.38	13.19	–	−.08	−.22**	.01
				[−.17, .02]	[−.31, −.13]	[−.09, .10]
5. education level	–	–	–	.07^+^	.18**^+^	.02^+^
				[−.03, .16]	[.09, .27]	[−.07, .12]
Leaders						
1. health-oriented leadership	5.27	1.27	.89			
2. self-rated health status	4.05	1.01	–	.28**		
				[.09, .45]		
3. job satisfaction	3.02	0.55	.86	.41**	.42**	
				[.23, .56]	[.24, .56]	
4. age	42.03	11.78	–	.06	−.22*	.20*
				[−.13, .26]	[−.40, −.02]	[.00, .38]
5. education level	–	–	–	−.08^+^	.12^+^	−.06^+^
				[−.27, .12]	[−.08, .31]	[−.25, .14]

*Note. M* and *SD* are used to represent mean and standard deviation, respectively. Values in square brackets indicate the 95% confidence interval for each correlation. The confidence interval is a plausible range of population correlations that could have caused the sample correlation (Cumming, 2014). * indicates *p* < .05. ** indicates *p* < .01. ^+^Cases in which Kendall’s Tau is reported instead of Pearson correlation coefficient because of the ordinal scale of the variable *education level*

Thus, significant correlations between HoL and health status or job satisfaction were found for the data set part of the leaders and the employees. H1a and H1b assumed that HoL (staff-care) is positively related to the health status. To test the relationship, we carried out a linear regression for each case. H1a (employees) was supported, with a moderately positive effect size (β = .40, *p* < .001) [77]. Here, HoL explains 16% (*R*^*2*^_*adj.*_) of the variance (*F*(1, 441) = 85.11). H1b (leaders) was supported with a weak, positive effect size (β = .26, *p* < .001) [77]. HoL explains 6% (*R*^*2*^_*adj.*_) of the variance (*F*(1, 103) = 7.41).

**Fig. 2:**
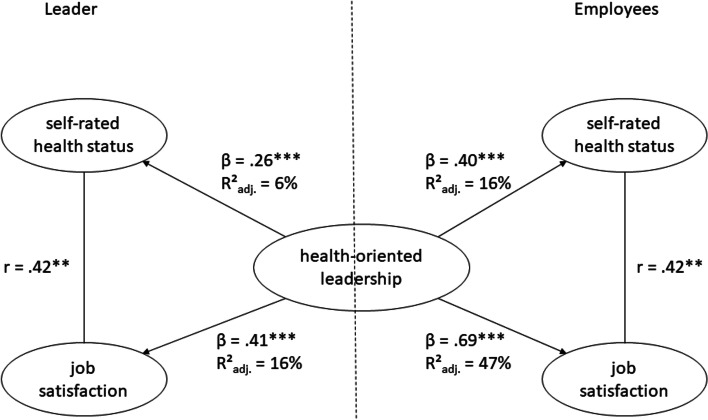
Results of hypotheses 1 and 2. *Note*. * indicates *p* < .05. ** indicates *p* < .01 *** indicates *p* < .001

In H2a and H2b, we expected that HoL is positively related to job satisfaction. Among employees (H2a), we found a significant relationship of HoL and job satisfaction with a strong, positive effect size (β = .69, *p* < .001) [77]. HoL explains 47% (*R*^*2*^_*adj.*_) of the variance (*F*(1, 420) = 380.9). The data set of leaders (H2b) also showed a significant relationship of HoL and job satisfaction with a medium to strong positive effect size (β = .41, *p* < .001) [77]. HoL explains 16% (*R*^*2*^_*adj.*_) of the variance (*F*(1, 100) = 19.85). Thus, H2a and H2b were supported. All relationships (H1 and H2) can be found in Fig. 2.

In H3, we tested in the leader sample if female leaders show higher HoL (staff-care) than male leaders. The t-test showed no significant difference between the groups (*M*_*male*_ = 5.09, *SD*_*male*_ = 1.28, *M*_*female*_ = 5.49, *SD*_*female*_ = 1.22, *t*(103) = − 1.55, *p* = .06), with a test power of 93%. These findings provided no support for H3.

H4 postulated that employees assess the HoL (staff-care) of female managers higher than of male managers. The performed t-test found no differences (*M*_*male*_ = 4.09, *SD*_*male*_ = 1.73, *M*_*female*_ = 4.24, *SD*_*female*_ = 1.81, *t*(439) = − 0.90, *p* = .18), with a test power of 58%. Thus, our findings did not provide support for H4.

To test H5a and H5b, we divided the employee sample in female (H5a) and male (H5b) leaders. For H5a we assumed that female employees assess the HoL (staff-care) of a female manager higher than male employees. For H5b we postulated that male employees assess the HoL (staff-care) of a male manager higher than female employees. Neither our findings in the employee data set of female managers (*M*_*male*_ = 4.32, *SD*_*male*_ = 1.69, *M*_*female*_ = 4.22, *SD*_*female*_ = 1.84, *W* = 2536, *p* = .55, test power of 6%), nor in the employee data set of male managers (*M*_*male*_ = 4.09, *SD*_*male*_ = 1.73, *M*_*female*_ = 4.09, *SD*_*female*_ = 1.74, *t*(261) = − 0.005, *p* = .50, test power of 5%) provided support for H5a or H5b. To investigate if leaders’ gender could be a moderator on the relationship between HoL and job satisfaction or self-rated health, a simple moderator analysis was performed using PROCESS. The outcome variables for this analysis were (1) job satisfaction and (2) self-rated health status. The predictor variable for the analysis was HoL. The moderator variable evaluated for the analysis was leaders’ gender. The interaction between HoL and leaders’ gender on job satisfaction (1) was found to be not statistically significant (95% C.I. [− 0.002; 0.095], *p* = .06), as well as the interaction effect between HoL and leaders’ gender on self-rated health status (2) (95% C.I. [− 0.076; 0.166], *p* = .46). These results identify leaders’ gender as a non-moderator of the relationship between HoL and (1) job satisfaction or (2) self-rated health status.

## Discussion

The aim of this study was (1) to explore the relationships between HoL and the health status assessed by employees and leaders, (2) to analyse the relationships between HoL and the job satisfaction as a non-health-related outcome for employees and leaders and (3) to examine differences in the assessment of HoL between men and women in a representative dataset of the working population in Germany.

Consistent with the first set of hypotheses, we found a relationship between HoL and the general health status for employees (H1a) and leaders (H1b) as well as a relationship between HoL and the job satisfaction for employees (H2a) and leaders (H2b). Regarding H3, H4, H5a and H5b no significant differences in the assessment of HoL between men and women were found.

Existing research already indicated the relationship between HoL and health-related outcomes like the general health status for employees [26, 27]. The respective hypotheses (H1a) turned out to be significant, for employees with higher effect sizes than shown in other studies. Moreover, in line with scarce previous research [1, 54], HoL assessed by leaders is related to the general health status of leaders as well (H1b).

Only little research has been conducted in the research-field of job satisfaction as a non-health-related outcome variable of HoL in the past. Few studies had already shown a relationship between the job satisfaction and staff-care or a buffering effect of staff-care from job demands to job satisfaction for employees [1, 13, 31]. To the best of our knowledge, previous research reported effects on employees rating of job satisfaction, but not on leaders’. We found both, a significant relationship between HoL (staff-care) and job satisfaction by the rating of employees (H2a) and leaders (H2b).

Some studies [33, 34] pointed out that surveying and looking at the genders of leaders and employees in the context of healthy leadership is important and should be further explored. We found no significant results for the tested hypotheses (H3, H4, H5a, H5b). For both, manager and employee ratings, no significant differences were found for assessing HoL (H3, H4). These findings do not support the postulated assumptions, which were based on previous evidence [1, 27, 33, 34, 67, 68]. The hypothesized assumptions that female employees assess the HoL (staff-care) of a female manager higher than that of a male manager (H5a), as well as the assumption that male employees assess the HoL (staff-care) of a male manager higher than that of a female manager (H5b) can also not be supported. This also deviates from the results found by Vincent (2012) [33] and are more in line with the results of Pundt and Felfe (2017), who found no significant differences in HoL related to gender [1].

In the case of the non-significant results presented here (H3, H4, H5a, H5b), it is debatable whether there is indeed no gender difference in HoL or whether the difference just could not be found. First, one could argue that due to a small sample size it could be possible, that no effect has shown up. Sometimes, results based on small sample sizes are criticized whether statistical significance can be claimed effectively [78]. Lantz (2013) points out that “Small samples do not make statistically significant results less statistically significant.” (p. 488, [79]). To check this circumstance, we take a closer look at the power analysis which was performed. For H3, we can state that with a power of 93% significant differences should have been found. Moreover, we must consider, that the *p*-value of .05 was only narrowly missed, indicating that a difference in the population, while not entirely improbable, was not evident in our sample. Regarding H4 (58%), H5a (6%) and H5b (5%), this assumption can not be made. The respective groups (divided into female and male managers) were probably too small overall.

Regarding the non-significant moderator-analysis of HoL on job satisfaction, it could be stated that the *p*-value of .05 was only narrowly missed. This may be due to different characteristics of the subsets (male and female employees). In the subset of female employees, 60% were male managers, while in the subset of male employees they counted 83%. Overall, it could also be possible that the sample was not large enough (*n* > 1000) to show significant effects.

When identifying gender differences, contextual factors such as company size, industry and hierarchical levels also have a structural influence on the proportion of female managers [34]. It is also possible that the attribution of HoL is influenced by gender stereotypes and that this creates the impression that women are more inclined to the issues than men are [80]. A similar statement was also made by Pundt and Felfe (2017). Accordingly, higher ratings in HoL (staff-care) reflects the women gender role. Another attempt to explain our non-significant results could be as follows: If we assume that the female managers in our sample did not exhibit a “traditional” female gender role (or Traditional Gender Role Belief [TGRB]) but rather had a more male role-model to get into a manager-position, it would not be surprising that no effects could be found. Since we did not survey TGRB, we cannot conclusively resolve this in a plausible way. Referring to a study of Elprana et al. (2015), women tend to have a lower affective Motivation to Lead (a-MtL) [23], and thus the proportion of women in manager-positions is even lower than men. Regarding the sample of the leaders, 37.9% of the managers were female. This could explain our non-significant results.

### Theoretical implications

Our study is another contribution to the leadership literature and adds a significant gain in knowledge to the current state of research on the relationship between HoL and job satisfaction, including an examination of gender differences in the level of HoL. Regarding the relationship between HoL and health status of employees, our study replicates existing research. For leaders’ we can also state a relationship between HoL and health, which also gains knowledge to the current state of research.

The findings also show that HoL is a much stronger indicator of job satisfaction than the health status. HoL would have been expected to be the stronger indicator of health status. A reason could be seen in the fact that health was only measured with a single item and the item does not necessarily measure or includes psychological health. If it is seen as a more general health measure which includes physical suffering, this maybe cannot be really addressed by leaders. Another reason could be that the workplace is obviously often about corresponding job satisfaction, turnover-intentions, and commitment rather than health status itself. Jiménez et al. (2017) showed [63] that HoL can provide resources and negative consequences of stress can be reduced. Therefore, more resources can lead to higher job satisfaction. However, it is possible, as already described by Bregenzer et al. (2020), that increased job satisfaction can be observed because managers contribute to a more comfortable work environment through HoL. This may result in an overall higher job satisfaction, related to individual aspects of the work environment [13]. Regarding the results of the leaders’ ratings of HoL and job satisfaction, the aforementioned argument could be reversed. One can argue that leaders who are satisfied with their job may have more resources and less stressors at work. Thus, it is easier for them to engage in healthy leadership. A recent study points in a similar direction: leaders not being strained were better able to engage in staff-care because they have more resources to foster employee health than strained leaders [31]. As the present study focused on staff-care, the self-care of the leaders’ may play a moderating role regarding the relationship between leaders’ assessment of HoL and their own job satisfaction. A closer application of the facet behaviour may help clarify these relationships.

### Study limitations and future research

There are also limitations of our study which are presented below. First, the use of cross-sectional data for testing relationships must be named. Since the current study is correlational, no causal assumptions can be drawn. As mentioned above, it could be possible that both, self-rated health status and job satisfaction, influence HoL, rather than vice versa. Previous research indicates that this seems to be unlikely and self-rated health status as well as job satisfaction are the outcomes of leadership [13].

Secondly, all study variables were measured on leaders’ and employees’ self-reports, which is known as common method bias and might have led to social desirability which affects the given answers. To avoid socially desirable responses, participants were assured strict anonymity [81]. The self-view of leaders could be biased because it might not represent the actual leadership behaviour.

Third, workplace outcomes like engagement, performance, turnover intention, as well as other health-related or psychological health parameters like wellbeing, stress or burnout/exhaustion were not included in the study but some studies already investigated these relationships [12, 24]. We suggest that future research could include traditional gender role beliefs or same-sex role models to identify other moderators. Regarding the non-significant moderator analysis of HoL on job satisfaction with managers’ gender as a moderator, it would be purposeful to achieve an even larger sample with balanced gender ratios in a follow-up study.

Another limitation results from the time at which the survey was conducted. Since there were effects due to the SARS-CoV-2 pandemic in June 2021, it is questionable to what extent HoL was influenced by this fact. As there is already evidence, that the effectiveness of HoL during crises increases [52] and by displaying staff-care, leaders can buffer negative effects of crises on followers [82], it would be interesting to record possible changes in the further course of or after the pandemic as longitudinal data, respectively.

Regarding the questionnaire, we used self-developed questions for the assessment of HoL. The factorial loadings were checked by an EFA and the item-total-correlations suggested a good fit with one factor, as well as good to very good reliability measures. Besides, from an occupational medical point of view, the questions worked out well. Nevertheless, the questionnaire should be reviewed on factorial and content validity in a second survey. Furthermore, it is important to mention that the questionnaire, which was based on the HoL instrument of Pundt and Felfe (2017), does not cover the subarea of awareness. In the context of our project “Healthy working in Thuringia”, we aimed to apply the self-designed questionnaire for the use in small and medium-sized enterprises. There, the managers were already informed about the facet awareness and we deliberately focused on the aspects of behaviour and value. However, a redesign of the questionnaire with the facet awareness should be considered.

### Practical implications

In today’s fast-changing world of work with ongoing digital stress, leadership and maintaining health is more important than ever. Organisations want healthy employees who are satisfied at work and do not develop turnover intentions. Therefore, organisations should continue to invest in occupational health management. In addition to occupational safety and health, which is regulated by law in many countries, organisations should also invest in workplace health promotion, based on behavioural and situational interventions. The approach of HoL could be communicated in management trainings, taken up in interventions for employees (e. g., salutogenic approaches for strengthening resources) and thereby anchored in the corporate culture for the long term.

Regarding the absence of gender differences assessing HoL, this finding leaves a wide scope in the implementation of health-promoting measures. Management training courses, for example, could be a suitable way to put these scientific findings into practice.

## Conclusion

On the one hand, we found significant effects of HoL on health status and job satisfaction, among both, employees, and managers. On the other hand, unlike previous studies [1, 33, 34], we summarize that there is no gender difference regarding HoL. Thus, we conclude that HoL is of great importance for organisations in terms of health and job satisfaction and could be implemented in management coaching and consulting sessions for organisations. Regarding gender, management training courses do not need to be specifically tailored to it. However, this also means that these scientific findings should be taken up in this type of coaching and that equal treatment should be focused here.

## Data Availability

The datasets generated and analysed during the current study are not publicly available due to German national data protection regulations but are available from the corresponding author on reasonable request.
